# Constructing and contesting industry’s role in multistakeholder governance: a qualitative analysis of responses to WHO consultations

**DOI:** 10.1186/s12992-025-01159-8

**Published:** 2025-11-11

**Authors:** Amber van den Akker, Xinmei Sun, Britta K. Matthes, Kathrin Lauber, Anna B. Gilmore

**Affiliations:** 1 Department for Health, University of Bath, Bath, UK; 2 Department of Linguistics and English Language, Lancaster University, Lancaster, UK; 3 School of Social and Political Science, University of Edinburgh, Edinburgh, UK

**Keywords:** Multistakeholder governance, Global health policy, Global governance, Corporate capture

## Abstract

**Background:**

Multistakeholderism as a norm stating that global public issues should be addressed by all those who affect or are affected by this issue, has become increasingly institutionalised in global governance, including the United Nations (UN) system. Despite an increasing body of evidence showing the risks of corporate capture of multistakeholder governance (MSG) and its related inability to deliver effective public health outcomes, this approach is increasingly common. While research shows that industry actors have pushed for MSG, and others have questioned its legitimacy, *how* MSG is constructed, legitimised and contested by different actors has not been systematically studied. Analysing responses to World Health Organization (WHO) consultations related to non-communicable diseases (NCDs) and associated risk factors, this study examines how actors construct or contest the legitimacy of MSG to address these public health issues.

**Results:**

Our analysis of 135 responses to 10 consultations revealed significant differences in how actors justified or contested MSG. Proponents of MSG, primarily industry-affiliated organisations, often cited industry expertise and resources as key justifications for a multistakeholder approach, in which they invariably included industry as a key stakeholder. Conversely, non-profit and academic respondents often argued against industry inclusion in MSG, referring to conflicts of interest, corporate capture of the policy process and the associated risks that MSG with industry actors poses to democratic and effective policymaking. While actors commonly invoked the same values (e.g., participation, effectiveness, fairness), they interpreted these differently, to argue for or against the inclusion of industry in MSG.

**Conclusion:**

Our findings underscore the contested nature of MSG, with actors calling on similar values and terminology to support fundamentally opposing positions. The ambiguity of concepts like participation, accountability and conflicts of interest may risk creating an opportunity for private sector interests to promote MSG as an ambiguous governance concept to fit their agenda. Given the normative role of WHO and other UN agencies in shaping what is considered ‘good governance’, it is essential for the WHO and other UN agencies to critically examine the evidence on multistakeholder approaches before deciding on its appropriateness and use unambiguous terminology when discussing their interpretation of the approach.

**Supplementary Information:**

The online version contains supplementary material available at 10.1186/s12992-025-01159-8.

## Background

Multistakeholderism as “a global norm specifying that global public problems *ought* to be addressed by those actors who *affect*, or are *affected by*, these problems” (emphasis in original) [1, p. 356], has increasingly taken hold in global governance institutions since the 1990s, to the extent that it is now “embedded in the fabric of global governance” [2, p. 257]. While there are variations in their composition, procedures and rules, the rise of multistakeholderism reflects a fundamental shift in norms of what practices constitute ‘good’ governance and who should govern instead [1, 3]. As evidenced by a growing literature base, the reliance on multistakeholder governance (MSG) opens up space for commercial actors in particular to influence the policy-making process [4–6].

A commitment to partnership and MSG facilitates the inclusion of different actors in the policy process and so enables corporate influence on policy [7, 8]. At the national level, a rapidly growing body of evidence shows the repeated inability of MSG approaches to develop effective public health responses to NCDs [9, 10]. Research shows how taking a multistakeholder approach to policy leads to less stringent targets, that are more likely to be voluntary and in line with industry narratives around individual responsibility and a focus on consumer education rather than addressing the more structural drivers of NCDs [11–13]. In some instances, this dilution is a direct result of adhering to a collaborative policy process, as the desire to maintain a collaborative, multistakeholder governance approach supersedes stringent regulation if this threatens to disrupt collaboration [5].

At the global level, some UN agencies have expressed concerns about multistakeholder platforms, with UNICEF, for example, stating that ultra-processed food (UPF) companies can use multistakeholder platforms “as conduits for their influence” [14]. A 2022 flagship report of the Food and Agriculture Organization (FAO), co-authored with other international organisations including UNICEF and WHO, similarly warns that the ‘multi-stakeholder model’ might further increase the power of the private sector and enable its influence on food policy and regulatory processes [15]. At the same time, other parts of the UN system (including other parts of the FAO) have been less hesitant, as evidenced by SDG17 explicitly being about ‘partnerships for the goals’ [16], as well as by UN agencies publishing guidelines for building ‘high-impact’ partnerships [17].

And yet, despite objections to MSG due to its perceived enabling of ‘corporate capture’ of public processes [18–20], and related harmful implications for democracy and trust in public institutions [21, 22], multistakeholderism continues to be promoted at the highest levels of governance as a way forward for global governance [23, 24]. The use and endorsement of multistakeholder approaches by UN institutions such as the WHO has been noted as a key driver for national governments to adopt such multistakeholder approaches [25]. Research has highlighted the risks of corporate capture in policymaking [5, 18, 26] and shown that MSG has been used to argue for the inclusion of industry [27, 28]. However, less is known about how MSG itself has been legitimised as a governance approach at the global level, and who have advocated for its adoption.

The success of lobbying efforts is contingent on many different factors outside of the control of those seeking to influence the policy process [29]. In the context of the interconnected, multi-level nature of governance shaping NCD policies, actors may therefore strategically seek to influence multiple policy venues at the same time [30, 31]. Industry actors are known to strategically employ ‘venue shopping’ to circumvent or prevent national legislation [32–34]. Research across policy settings indicates that industry actors and groups representing business interests have more frequent, substantial and varied ways of engaging with the policy process at the national and global level, through either formal or informal, ‘backstage’ policy processes, when compared to other interest groups [7, 35–37]. This access to the policy process is used strategically to counteract or support specific policies, cast doubt on underpinning evidence, or seek to change the rules of the policy-making process itself [38–40]. Recognising the strategic use of argumentation in corporate influence, understanding how MSG is constructed, legitimised and contested could facilitate a more in-depth understanding of the increasing popularity of MSG across global and national governance institutions.

Addressing this gap, the present study aims to examine different stakeholders’ arguments around MSG and explore how they construct and/or contest it as a legitimate approach to address global problems. To do so, it will focus on the WHO – a UN agency with a significant normative role in shaping national government’s approaches to governing public health, and where MSG is increasingly prominent [41, 42] – and responses to NCD-related consultations – a site where public and corporate interests often clash.

## Methods

We approach MSG through the arguments used by multiple actors to either construct or contest its legitimacy. Adopting a focus on narratives is relevant, given that rather than being a ‘fixed’ part of global governance, multistakeholderism is, like other political institutions, continuously constructed, maintained and contested [1, 43]. Through this approach, we challenge the narrative that multistakeholderism has ‘emerged’ as a solution to problems of ineffectiveness and bureaucracy, instead recognising this as an active process of producing “problems” that multistakeholderism may or may not be the solution for. This allows us to probe the underlying assumptions that present or contest multistakeholderism as a legitimate ‘solution’ to global health ‘problems’ [43].

### Data collection

In February 2024, we collected responses to all WHO consultations related to NCDs and associated risk factors. A follow up search was conducted in September 2024. A list of consultations was obtained from the WHO website and through the first 100 results of a Google search. Any available consultation responses were downloaded from relevant WHO webpages (https://www.who.int/news-room/articles, https://www.who.int/news-room/events). Where consultation responses were not publicly available, we requested them via email from the WHO.

To identify relevant submissions among the retrieved consultations responses, we started by using a search query (multistakeholder* OR multi-stakeholder* OR public-private partnership* OR ppp* OR partner* OR collaborat*) which contained terms commonly used in relation to MSG. After two authors (AvdA and XS) closely read a sample (*n* = 20) of documents which discuss MSG, we extended our search query to include other terms mentioned in the context of MSG (“multiple stakeholders” OR “various stakeholders” OR “all stakeholders” OR “all relevant stakeholders” OR engag* OR multisector* OR multi-sector*). The search term engag* aimed to capture phrases such as “engagement with the private sector”. We also included ‘multisectoral’, recognising that while often used to refer to sectors of government, it is sometimes used interchangeably with multi-stakeholderism [44].

To be included, a response had to contain any of the abovementioned search terms in the main text. We excluded duplicate submission. Given the focus on arguments to (de-)legitimise MSG, we also excluded responses that only referred to MSG in a descriptive way, for example, mentioning a multistakeholder platform.

All included responses were imported into NVivo 14 (Lumivero) for analysis, noting the year, consultation topic (e.g., alcohol, diet, physical activity, NCDs) and type of respondent. Supplementary file 1 presents the categorisation of respondents.

### Analysis

We first conducted a descriptive analysis of the consultation responses to map the use of different terminology around MSG and its legitimacy. To guide our analysis we drew on a typology that proposes that the legitimacy of global governance institutions can be justified by arguments related to democratic, technocratic and fair reasoning, applied to either the procedure or the performance of the global institution (see Table 1) [45]. Legitimacy in its broadest sense can be defined as the perception that an organisation has a right to rule and exercises it appropriately [46]. A global governance institutions’ legitimacy is continuously being shaped, maintained and contested through the statements these institutions make and the reasons they give to justify their right to rule [47]. The typology developed by Scholte and Tallberg (2018) builds on a rich body of literature on global governance legitimacy, with roots in Weber’s three core grounds of sociological legitimacy (rationality, tradition and charisma) [48] and Habermas’ approach to legitimacy as derived from effectiveness [49]. More recent theoretical contributions have posited that governance institutions are legitimate insofar as they are perceived to be predictable, justifiable, equitable, accessible and respectful of human dignity [50]. When examining processes of legitimation or de-legitimation in global governance institutions, a distinction is made between different phases of the policy process where legitimacy can be gained or lost. Scharpf (1999) first distinguished between process (the way decisions are made) and substance (the result of decisions) in the case of EU authority [51]. Schmidt (2013) further added ‘throughput’ legitimacy as a separate stage for consideration [52]. The distinction made by Scholte and Tallberg (2018) between ‘procedure’ and ‘performance’ builds on this distinction between input, output and throughput. Rather than separate, procedural and performance sources of legitimacy may build on one another and coexist [45].

Hurd (2008) proposes “favourable outcomes”, “fairness” and “correct procedure” as grounds for legitimacy [53]. Scholte and Tallberg (2018) expand and further systematise this operationalisation, distinguishing in their typology between legitimacy justifications based on democratic, technocratic and fair values, each of which can be applied to both the procedure and/or performance of a global governance institution [45]. Recent research supports this distinction, showing how global governance institutions themselves use these three types of justifications to legitimise their role [54].

We applied the framework by Scholte and Tallberg to examine justifications to either legitimise and further institutionalise – or contest the institutionalisation of – multistakeholder governance. We then used an inductive approach to draw out *how* the arguments for these justifications were made by different actors. We coded at the document level, with some documents being coded multiple times when they employed multiple arguments.

Three members of the research team (AvdA, KL and BM) met to discuss and pilot the coding framework. We independently read and coded four texts to the typology and afterwards we discussed any discrepancies or uncertainties in interpretations of the framework as they came up. Through in-depth open discussion we reached a shared understanding of the framework in relation to our dataset. Next, the lead author (AvdA) coded the remaining documents and regularly met with KL and BM to discuss codes and illustrative quotes as well as any uncertainties, to ensure that we were still in agreement over how the framework was used. Any disagreements were discussed until consensus was reached.

**Table 1:** Typology of the justifications for legitimacy of global governance institutions (GGIs) (Scholte and Tallberg, 2018)

	Democratic	Technocratic	Fair
Procedure	**Participation** All affected parties have due involvement in and deliberation around a GGI’s policymaking processes	**Efficiency** The number and speed of a GGI’s policy decisions	**Impartiality** Policymaking processes are followed consistently and without discrimination
	**Accountability** A GGI through transparency, consultation, review and redress adequately answers to the publics that it affects	**Expertise** Basing policy decisions on the best knowledge and skills	**Proportionality** Members contribute to GGI’s resourcing in accordance with their relative means
Performance	**Democratic performance** When a GGI’s activities increase popular participation and public accountability in wider society	**Problem solving** The fullest and fastest realisation of results	**Human dignity** The outcomes uphold norms of basic humanity for all
		**Collective gains** The largest benefits for society	**Distributive justice** The benefits are equitably shared among those concerned

## Results

### Overview of consultation responses

We collected 784 responses related to 15 consultations. The responses to the remaining 25 consultations were not publicly available. We obtained an additional 420 responses to one consultation from the WHO. Of the collected 1,204 consultation responses, 181 contained at least one of the search terms. Of those 21 were excluded as duplicates and 25 were excluded as only descriptive. 135 (11.2%) were included in the analysis. These stemmed from 10 consultations that took place in 2017 (*N* = 22), 2018 (*N* = 1), 2020 (*N* = 52), 2021 (*N* = 32), 2022 (*N* = 24) and 2023 (*N* = 4). Supplementary file 2 presents an overview of the included consultations, where they were obtained and the number of responses that were included in this study. The majority of responses including in our analysis were from NGOs (*N* = 59, 43.7%), followed by trade associations (*N* = 37, 27.4%), national governments (*N* = 14, 10.4%) and multi-stakeholder platforms (*N* = 11, 8.1%).

### Terminology used to discuss multistakeholder governance

The mostly commonly used term(s) were ‘multistakeholder*‘ or ‘multi-stakeholder*‘ (25.7% of total number of terms used), ‘multisector*’ or ‘multi-sector*’ (23.9%), ‘all stakeholders’ or ‘all relevant stakeholders’ (15.5%) and ‘public private partnership*’ or ‘ppp*‘ (14.4%) (See Table 2).

**Table 2:** Frequency of terms used in consultation responses, by actor type

Actor category	No. of responses with the search term(s) (total no. of terms used)	Frequency of terms across responses								
		multistakeholder* or multi-stakeholder*	“multiple stakeholders”	“various stakeholders”	“all stakeholders” or “all relevant stakeholders”	public-private partnership* or ppp*	partner* with	collaborat* with	engag* (with/of)	multisectoral or multi-sectoral or multi-sector
Academic	6 *(16)*	4	0	0	0	3	0	0	6	3
Corporation	3 *(9)*	6	0	0	1	0	0	0	0	2
Multi-stakeholder platform	11 *(42)*	12	0	0	7	0	1	0	8	14
National government	14 *(45)*	17	0	0	1	1	0	0	5	21
NGO	59 *(146)*	20	2	5	29	16	10	1	10	53
Public health agency	3 *(3)*	0	0	0	0	0	0	0	0	3
Think tank	2 *(3)*	0	0	0	0	1	0	0	0	2
Trade association	37 *(181)*	55	2	5	31	43	7	3	26	9
Total	135 *(444)*	114	4	10	69	64	18	4	55	106

### Justifications for and against a multistakeholder approach

A wide range of arguments were used for and against MSG which could be mapped to all categories in the typology (Table 1). Most arguments centred around the in- or exclusion of industry actors in MSG, and the democratic, technocratic and fairness considerations associated with this. Table 3 provides an overview of the key arguments used for and against MSG in each of the topic areas covered above.

**Table 3:** Key arguments used for and against MSG, categorised by typology

Topic area in typology	Pro-multistakeholderism	Against multistakeholderism
Democratic	• Participation explicitly include industry.	• Not all non-state actors are the same; industry should be excluded. • Multistakeholder governance poses a threat to democracy.
Technocratic	• Multistakeholder governance enables efficient partnership processes. • Industry expertise and resources are needed for effective policy, which should mostly be ‘fixes’ industry can do.	• Collaborative processes may support efficient policy processes. • Multistakeholder governance contributes to legitimisation of industry inclusion in policy, which is a main barrier to effective policy-making.
Fair	• All stakeholders should be treated equally - industry should not be unfairly ostracised through conflicts of interest policies.	• Strong conflicts of interest guidelines needed to prevent undue industry influence. • Protecting human rights is key priority of states and international organisations, and multistakeholder governance threatens their ability to do so.

In the following, we present key findings by each of the domains outlined in Table 1.

#### Arguments centred around democratic procedure and performance

**Participation** was the most common justification for adopting a multistakeholder approach, mentioned in 61.5% of the responses. However, there were significant differences in who respondents argued should participate in MS initiatives: NGO and academic organisations often explicitly excluded the private sector. For example, an NGO noted that *“the benefits of multisectoral approaches to alcohol harm are substantial”* while later emphasising that

*we must highlight that all stakeholders in WHO GAS [Global Action Strategy] are not equal […] the alcohol industry should not be placed in equal standing with international partners and civil society as the current working document does. The alcohol industry is the single biggest obstacle to WHO GAS implementation around the world.* (NGO)

In contrast, industry-affiliated respondents repeatedly and explicitly emphasised private sector inclusion, with one trade association, for example, noting that *“We believe that partnership working is fundamental to reducing harmful drinking and must include all relevant stakeholders*,* including the industry”*. However, those advocating in favour of multistakeholder or multisectoral governance often failed to – or purposefully did not – clarify which ‘stakeholders’ or ‘sectors’ they referred to.

Arguments related to **accountability** of multistakeholder processes featured in 25.2% of consultation responses, though generally not by those critical of MSG. Many of those in favour of multistakeholder action recognised the need for accountability and mostly suggested transparency mechanisms or relying on trust relationships. As one government respondent suggested,

*COI mitigation and management tools*,* such as Codes of Conduct*,* can be used to increase transparency and reduce risk around actual COIs while promote successful multi-stakeholder partnerships. Codes of Conduct*,* non-binding sets of principles for engagement among stakeholders*,* promote transparency and accountability. (*national government*)*

This was similarly expressed by many industry-affiliated actors, suggesting that *“disclosure plus transparency can be the main tools to limit influence not aligned to mission.”* (consultant).

While arguments around **democratic performance** were not common, used in 7.4% of responses, almost identical arguments related to this were utilised both as arguments for and against MSG. One industry-affiliated respondent argued that “*Unjustifiably restricting engagement with the private sector would not only be antithetical to democracy and good governance*,* it would also deprive member states of the knowledge*,* expertise and resource of the private sector”* (trade association). At the same time, some respondents opposing MSG used very similar arguments, noting *“the risks that this [multistakeholder governance] model brings to […] democratic policy processes”* and arguing that MSG had *“CoI* [conflicts of interest] *intrinsically built in and erodes decision-making in public interest* [sic]” (NGO). WHO’s engagement in multistakeholder partnerships to date, for example the WHO Global Coordination Mechanism, was seen to further legitimise the multistakeholder governance approach, as it *“possess[es] ‘normative’ impact and value and hence provide[s] non-state actors with further global legitimacy”* (NGO).

#### Arguments centred around technocratic procedure and performance

Arguments of **procedural efficiency and technical expertise** were used in 27.4% and 30.4% of responses respectively, being particularly highly prevalent in the responses from industry-affiliated actors, especially when justifying the inclusion of private sector actors. Industry’s ‘resources’ and ‘expertise’ were argued to be crucial not only for the success of the policy response in question, but also that of the WHO itself. For example, one trade association stated that

*By seeking greater input*,* not less*,* global institutions can best aid in the development of strong*,* sustainable and widely supported outcomes that are built on a science- and evidence-based approach. Efforts to restrict the ability of member states to engage with stakeholders*,* particularly in the private sector*,* groups [sic] can only result in weaker policy outcomes and undermine the credibility of not only the final policy recommendations but also the institutions that recommend them. (*Trade association*)*

The emphasis was placed especially on industry’s ‘technical expertise’ and the necessity for drawing on this expertise to enable specific policy options:

*During the policy development process*,* the role of the private sector in helping to inform science-based policy development and assisting in implementation*,* drawing on our breadth of technical expertise (e.g.*,* in product reformulation) to help people everywhere achieve and maintain healthy diets (*Trade association*)*.

Notably, the specific policy options that these respondents suggested for multistakeholder working predominantly focused on ‘problematic consumption’, ‘product reformulation’ and ‘behaviour change’ and they mostly advocated for technological ‘fixes’ to public health problems. A trade association in the alcohol sector for example noted that it has *“committed itself to a variety of actions to stimulate responsible drinking”* and that they *“work together with all other stakeholders to promote responsible alcohol consumption”*. Similarly, different trade associations in the food industry noted the private sector’s *“breadth of technical expertise (e.g.*,* in product reformulation) to help people everywhere achieve and maintain healthy diets”*, as well as the *“essential need for multi-stakeholder partnerships […] to drive the reduction of nutrients of public health concern in the food supply or to drive change in consumer habits”*. An academic, who was critical of industry involvement in MSG, was the only respondent who explicitly included a warning to WHO regarding this approach, stating that:

*there are risks that conflicts of interest will compromise objectives and values of nations other than their public health goals […] The manufacturer wants the government to address the nutritional problem with an approach that would rely on using the manufacturer’s products. The government program*,* on the other hand*,* can meet its nutritional goals in multiple ways and might well achieve a better result using an approach that was not in the interest of the manufacturer. (*Academic)

Aside from the suggested benefits of industry inclusion for the quality of policy solutions, respondents in favour of MSG also referred to enhanced efficiency and cost-effectiveness of the policy process. For example, one trade association noted that multistakeholder approaches were necessary to allow them to *“join WHO in our willingness to promote evidence-based and cost-effective interventions.”* (Trade association). In response to WHO’s development of a conflict of interest tool, many respondents in favour of MSG argued that such a mechanism would limit the efficiency of the policy process, stating that *“Use of the tool in its entirety would require exceptional amounts of time and attention*,* which would both misuse limited resources and slow or effectively freeze the process of partnership”* (trade association).

Conversely, many NGOs and academic organisations warned against MSG as leading to less effective policy due to the risk of industry influencing the policy-making process, citing the industry’s *“record of undermining effective policy development”* (Academic), leading *“government resolve to bring in effective legislation [to] be undermined and global and national policy agendas subverted”* (NGO). This was directly linked to the MSG model, further noting that the industry “*bring[s] into play the powerfully promoted MSI and PPP model to position themselves as responsible ‘corporate citizens’ who should be seen as ‘a part of the solution’ – not a party to be regulated”* (NGO). Some of those in favour of multistakeholder approaches invoked partnerships they have had in the past as justifications of the **problem solving** ability of multistakeholderism, which was referred to in 34.8% of responses. Predominantly industry-affiliated respondents provided a list of ongoing or completed multistakeholder partnerships they themselves had been a part of to justify their advocating for multistakeholder approaches, though often without presenting evidence on their effectiveness from independent evaluations, only stating, for example, that *“these [collaborative] efforts have been a fundamental component of national government efforts to achieve public health goals. Without such collaboration*,* these goals would not have succeeded.”* (Trade association).

Conversely, those against industry involvement through MSG rejected these claims of effectiveness, noting that *“We identify no evidence of efficacy for continuing dialogue with the alcohol industry and deeply regret the disproportionate attention given to economic operators in the plan”* (NGO). Similarly, another NGO argued that

*In the action plan*,* the alcohol industry should be dealt with in a single paragraph*,* emphasizing that neither self-regulation*,* nor corporate social responsibility has brought any positive changes to the alcohol burden; that the alcohol industry is interfering against WHO-recommended alcohol policy solutions*,* delaying*,* derailing and destroying attempts to implement the WHO GAS. (*NGO*)*

By this, it clearly contesting the suggested efficiency and effectiveness of industry inclusion through MSG.

#### Arguments centred around fair procedure and performance

The main arguments related to **fair procedure**, referred to in 51% of responses centred around conflicts of interest (COI) and indicated strong disagreement between respondents. Some NGOs and academics referred to the ‘*inherent*’ COI between some industries and public health as a key rationale for not including industry actors in MSG. One NGO, for example, noted that

*the alcohol industry has a clear conflict of interest in public health policy settings due to the health impact of its products and reflected in its financial and legal obligations to shareholders. Therefore*,* alcohol industry representatives have no place in the formulation or enforcement of policies to reduce alcohol harm.* (NGO*)*

On the other hand, some respondents perceived COI tools as enabling MSG, with one member state for example suggesting that *“COI mitigation and management tools*,* such as Codes of Conduct*,* can be used to increase transparency and reduce risk around actual COIs while promote [sic] successful multi-stakeholder partnerships.”* (State). However, exactly this was deemed a risk by others, summarised by one NGO as

*a tension between an effort to try and safeguard policy and programming endeavours in nutrition and a simultaneous reaffirmation of the MSI/PPP [multistakeholder initiative / public-private partnership] paradigm. However*,* this paradigm has CoI intrinsically built in and erodes decision-making in public interest. […] Redefining of the CoI concept to serve the MSI/PPP paradigm can lead only towards further undermining rather than much needed strengthening of CoI safeguards.* (NGO)

In relation to **human dignity or distributive justice**, which were referred to in 4.4% and 7.4% of responses respectively, notably not by those in favour of MSG. Some NGOs emphasised the obligation of states and public institutions to protect human rights, using this narrative to problematise industry’s involvement through MSG. As one NGO argued, *“industry players have capitalised on the notion of public private partnerships and have consistently positioned themselves as contributors to development and job creation despite the impact of the production process on natural resources in countries”* (NGO). This aligns with another respondent’s assertion that the *“further entrenchment of the MSI/PPP model disregards the risks this model brings to human rights”* (NGO).

## Discussion

Through analysing the arguments used to construct or contest the legitimacy of multistakeholder approaches to global public health governance, this study highlights differences not only in how multistakeholderism was justified but also the specific strategies used to do so. Drawing on the framework by Scholte and Tallberg (2018), we noted that most justifications for MSG were based on democratic notions of participation, technocratic arguments of requiring expertise and resources from all sectors and arguments around the fairness of including or excluding predominantly commercial actors in MSG due to actual or perceived conflicts of interest.

Many of the arguments found in this study align with previous work on common arguments used in lobbying and corporate influence on policy [29, 39, 40]. These include arguments around the benefits of a multi-stakeholder approach to help reach public health goals in a democratic way and the feasibility of adopting multi-stakeholder approaches – or the non feasibility of not adopting a multi-stakeholder approach – to enhance the effectiveness or efficiency of the policy process [39]. However, while many private sector-affiliated actors referred to the need for private sector expertise and resources to be able to implement the, mostly technologically-driven and individually focused, policy solutions they proposed, they generally did not provide evidence to support the claim that multistakeholder approaches were able to deliver more effective and efficient policy results. Fairness arguments were mostly used in relation to conflict-of-interest policies and conflicting ideas about the accountability and protection of policy processes. References to fairness arguments were often countered by the exact opposite argument, with one side appealing to the unfairness of including powerful industry actors, and the other side countering this with the unfairness of excluding the same actors. This is supported by prior research showing that appeals to social values are often countered by opponents’ appeal to a conflicting value [29].

Notably, our findings highlight how ‘multistakeholder governance’ and supporting notions of ‘participation’ and ‘the stakeholder’ are used by different actors with fundamentally different – at times opposing – meanings and purposes. While all respondents included in this study referred to multistakeholder collaboration, they often did not explicitly state who they suggested should be in- or excluded in this collaboration. When they did, there were opposing views, with some arguing for industry inclusion as key stakeholder while others insisting on the exclusion of some industry actors as having inherent conflicts of interest. This indicates how MSG can be conceptualised as an “empty signifier”, i.e. a concept whose meaning is subject to interpretation and potential co-optation by different users [55].

Our results support previous work showing MSG has been promoted by industry actors seeking to portray themselves as ‘part of the solution’ [19, 27]. The strategic use of arguments about how the policy process ought to operate, and who should be involved as ‘stakeholders’, are a key way for corporate actors to exert long-term influence over the policy-making process [40]. Reviews of multistakeholder initiatives in the global food system repeatedly show that industry or industry-affiliated actors are among the most prevalent stakeholders in these initiatives [18, 56]. In this context, multistakeholder governance forums have been criticised for creating ‘pseudo-participatory forums’ that promise reforms and influence on decision-making processes to a range of actors and that, though the reforms may never materialise, may convince others to delay more radical action [57]. At the same time, multi-stakeholder platforms may effectively co-opt opposition, through creating a position of ‘the stakeholder’ that supposedly represent ‘civil society’ interests but who may in reality only put forward the most palatable – to the organiser of the multi-stakeholder initiative – position from the heterogeneity that makes up actual civil society [57, 58]. In this way, corporations may depoliticise dissent, engineer consent and divide opposition while amplifying or engineering the ‘stakeholder’ voices that reinforce industry-favourable narratives [59, 60]. Although this co-optation and depoliticisation of ‘stakeholders’ is a noted risk in the literature on MSG, the term was used uncritically, and often unexplained, in the documents we reviewed.

The emphasis on industry ‘expertise’ as a core constituent of MSG highlights a risk that multistakeholder governance approaches become a vehicle to drive industry-favourable narratives and solutions. MSG fundamentally relies on collaboration between actors whose interests and positions on both the problem and potential solutions may not align. The inclusion of different, sometimes adversarial, positions is arguably a core feature of democratic governance [61, 62]. However, while MSG does include different positions, its operational model often remains consensus-based, building on the assumption that different actors can and should reach a shared agreement on what the problem is and how it should be addressed [63]. There is a risk that MSG facilitates co-optation of the problem definition and proposed solution by powerful stakeholders, which in a consensus-based context may leave no other recourse for other actors but to assent or exit the process altogether [61].

An example of MSG enabling rhetorical co-optation was the 2021 UN Food Systems Summit, which was predominantly focused on technologically-derived solutions that were in line with industry narratives and highly dependent upon industry resources, rather than reflecting the breadth of problematisations and available solutions [18, 64]. Our findings similarly show that the use of technocratic arguments, predominantly by industry-affiliated actors, were mostly focused on ‘fixes’ that required industry resources, such as reformulation and consumer education, even though evidence repeatedly shows the limited effectiveness of such actions in the context of complex public health problems, which require fundamental social, political and economic transformations [65–67]. While the risk of this narrative drift towards technological solutions has been noted as a significant risk in the literature [20, 64], only one respondent in this study noted and warned against this as a potential risk with MSG.

We noted a tendency by some respondents to present MSG as good practice without further evidence to support this claim. Instead, references to MSG as ‘good governance’ were often underpinned by references to other international agreements that adopted or advocated for a multistakeholder approach, with SDG17, the 2021 UN Food Systems Summit and the Better Regulation agenda key among them. This is notable because on the opposing side, the ability of multistakeholder approaches to effectively address complex public health problems was heavily contested. The criticisms of MSG put forward by respondents are supported by evidence which shows that multistakeholder initiatives at national and global level have led to less effective solutions being proposed, which mostly rely on self-regulation and which were inconsistently adhered to [10–13, 68]. Moreover, multiple examples of national multistakeholder approaches to public health problems indicate that the solutions developed were not only less effective than regulatory approaches but that they were made less effective *because* of the multistakeholder process through which they were developed [5, 26, 37].

One of the few global multistakeholder platforms that has been independently evaluated, the UN Global Compact (UNGC), has been found to have significantly lower implementation of its principles than suggested through participants’ self-reporting [11]. In addition, UNGC’s members were more likely to show improvements on superficial dimensions while performing worse than non-members on more fundamental (and costly) dimensions [11, 69]. Notably, Berliner and Prakash (2014) further noted that a lack of monitoring and enforcement allowed members to shirk responsibility while enjoying the goodwill benefits of association with the UN [11], even as the integrity of the UN itself may be harmed by a loss of trust in a platform that they are seen to condone but which does not deliver on its promise [70]. This concern was echoed by some respondents in this study who similarly noted the risk that multistakeholderism may pose to the credibility of the WHO. Many respondents in favour of MSG predominantly referred to transparency and non-binding codes of conduct as key ways to establish accountability. Rather than asking how and to whom governance institutions should meaningfully be accountable when governing complex global issues, the emphasis on transparency and disclosure effectively depoliticises this issue, suggesting that being transparent about a process equates having accountability and that disclosing conflicts of interest is the same as adequately preventing and managing conflicts of interest, both of which do not do justice to the importance of adequately considering and managing these issues [22].

Lock and Davidson (2024) show how arguments used in lobbying may depend on the issue, the type of policy and the lobbying objective [39]. Beyond objectives directly related to the policy for which the consultation response was submitted, respondents referencing MSG appear to use these arguments with the objective to (de-)legitimise MSG as a *way of doing* policy. This occurs in a context of increasing institutionalisation of multistakeholderism as a goal in itself [1, 6, 71] and the use of multistakeholder venues to argue for further inclusion of industry actors and industry-favoured narratives in policy [18, 19]. Our findings show that these references to MSG as ‘good governance’ are predicated on prior use of the multistakeholder approach and anecdotal reports of these instances rather than independent evidence. In addition, the lack of clarity on what precisely is meant by multistakeholder governance and which ‘stakeholders’ this ought to include risks institutions seeking to adhere to MSG as ‘good governance’ in a way that allows those wielding the term to use it to justify their position or participation. There is a risk that MSG becomes the default because of its use in an increasing number of norm-setting reports and contexts, rather than due to any existing evidence of its success or critical examination of its appropriateness.

A key limitation of this study is that, as the WHO does not publish the responses to all their consultations, we were not able to search and collect the responses to 24 other relevant consultations. While consultation responses to the WHO is particularly relevant for public health policy, future work could meaningfully build on this by conducting similar analyses of MSG in the context of other UN bodies, some of which are more openly advocating for MSG. In addition, more in-depth historic analyses of MSG in UN bodies could be particularly useful in shedding light on the origins and spread of this concept, including who early adopters and promotors of MSG were.

## Conclusion

Through analysing the discourses used to justify or contest the legitimacy of multistakeholder governance, our findings show how different actors seek to construct or contest the legitimacy of multistakeholderism as a governance institution. Moreover, the findings show that these discursive struggles exist over not only the legitimacy of MSG but also the meanings of their underpinnings as the same justifications – at times the same terms – were used to argue for fundamentally different, at times directly opposing, approaches. Both those for and against MSG used arguments grounded in the democratic, technocratic and fair, although with different emphases. Crucially, however, different actors used the same justifications and terms but with different meanings to fit their paradigm stance, at times without making this explicit. This results in different actors attempting to wield the same terminology while meaning fundamentally different things, as for example with ‘participation’ or ‘conflict of interest’, which different actors use either in service to or against the multistakeholder paradigm. The risk here is that the terminology gets adopted by global governance institutions such as the WHO without clarity on these meanings. This may result in the perpetuating of potential misconceptions around MSG, as well as the presence of this terminology being used to justify a multistakeholder governance paradigm that is not based on evidence and which consultation respondents, despite using the same language, do not support. Given the importance of discourse in legitimising or delegitimising governance institutions, it is essential that the specific use of related terms is explicitly made clear. Moreover, given the norm-setting role that all UN agencies, including the WHO, have in steering global, national and subnational policy responses to public health, including the governance mechanisms through which these policy responses are to be arrived at, it is essential that the WHO guidance on and use of MSG is based on independent evidence and clearly described, to reduce the risk of co-optation of these terms to fit a specific governance agenda.

## Supplementary Information

Below is the link to the electronic supplementary material.

Supplementary Material 1

Supplementary Material 2

## Data Availability

The data that support the findings of this study are available from public websites of the WHO and respondents. Data related to one consultation is not currently publicly available.

## Abbreviations

COI: Conflict of interest
MSG: Multi-stakeholder governance
MSI: Multi-stakeholder initiative
PPP: Public-private partnership
SDGs: Sustainable development goals
UN: United Nations
WHO: World health organization
