# Supplementary material for: Constructing and contesting industry’s role in multistakeholder governance: a qualitative analysis of responses to WHO consultations
Source: Global Health. 2025 Nov 11;21:66. doi: 10.1186/s12992-025-01159-8 (PMC12606952; doi:10.1186/s12992-025-01159-8)
Supplement: Supplementary file 1 — Supplementary Material 1 [file 12992_2025_1159_MOESM1_ESM.docx]

**Supplementary file 1. Categorisation of respondents**

| **Actor category** | **Working definition** |
| --- | --- |
| Academic | Universities, research institutes and individual academics |
| Corporation | For-profit corporations, including transnational corporations |
| Multi-stakeholder platform* | An organisation that describes itself as a multi-stakeholder platform and includes member organisations from across sectors. |
| National government | Government and government departments/ agencies. |
| Non-governmental organisation (NGO)* | Non-governmental civil society organisations and social movements. |
| Public health agency |  |
| Think tank* |  |
| Trade association | An association that brings together multiple businesses |

*We note that a portion of multi-stakeholder platforms, NGOs and think tanks included in this study receive industry funding, are founded by or are otherwise affiliated with industry actors.
