# Supplementary material for: Constructing and contesting industry’s role in multistakeholder governance: a qualitative analysis of responses to WHO consultations
Source: Global Health. 2025 Nov 11;21:66. doi: 10.1186/s12992-025-01159-8 (PMC12606952; doi:10.1186/s12992-025-01159-8)
Supplement: Supplementary file 2 — Supplementary Material 2 [file 12992_2025_1159_MOESM2_ESM.docx]

**Supplementary file 2. Overview of consultations and number of included responses.**

| Consultation title | Year | Topic | No. of total responses | No. of responses included | Source |
| --- | --- | --- | --- | --- | --- |
| The scope of the guideline on polyunsaturated fatty acid intake | 2013 | Diet/nutrition | 15 | 0 | Google search query |
| The scope of the guideline on carbohydrate intake | 2016 | Diet/nutrition | 15 | 0 | Google search query |
| Safeguarding against possible conflicts of interest in nutrition programmes | 2017 | Diet/nutrition | 47 | 22 | WHO website, event page |
| The draft guidelines on saturated fatty acid and trans‐fatty acid intake for adults and children | 2018 | Diet/nutrition | 58 | 1 | WHO website, news page |
| Developing a Global action plan to reduce the harmful use of alcohol | 2020 | Alcohol | 251 | 52 | WHO website, news page |
| The scope of work for updating the FAO/WHO calcium, vitamin D, and zinc requirements in children aged 0–36 months | 2020 | Diet/nutrition | 22 | 0 | Google search query |
| The draft guideline on physical activity | 2020 | Physical activity | 420 | 0 | Provided by the WHO team |
| Final Evaluation of the WHO Global Coordination Mechanism for the prevention and control of Noncommunicable Diseases (GCM/NCD) | 2021 | Noncommunicable disease | 42 | 20 | WHO website, news page |
| The GCM/NCD Workplan 2022-2025 | 2021 | Noncommunicable disease | 17 | 6 | WHO website, news page |
| Recommendations to strengthen and monitor diabetes responses within national noncommunicable disease programmes, including voluntary global diabetes coverage targets for 2030 | 2021 | Noncommunicable disease | 46 | 6 | Google search query |
| The draft guideline on total fat intake for the prevention of unhealthy weight gain in adults and children | 2021 | Obesity | 24 | 0 | WHO website, news page |
| The draft guideline on carbohydrate intake | 2022 | Diet/nutrition | 19 | 0 | WHO website, news page |
| Updating Appendix 3 of the WHO global action plan for the prevention and control of noncommunicable diseases 2013–2030 | 2022 | Noncommunicable disease | 73 | 16 | Google search query |
| The draft guideline on use of non‐sugar sweeteners | 2022 | Diet/nutrition | 44 | 4 | WHO website, news page |
| The draft guideline on policies to protect children from the harmful impact of food marketing | 2022 | Diet/nutrition | 46 | 4 | WHO website, news page |
| The draft guideline on fiscal policies to promote healthy diets | 2023 | Diet/nutrition | 65 | 4 | WHO website, news page |
